# Editorial: Extracellular Matrix for Cardiovascular Reconstruction

**DOI:** 10.3389/fcvm.2021.664803

**Published:** 2021-04-08

**Authors:** Steven Bibevski, Sharan Ramaswamy, Joshua Hutcheson

**Affiliations:** ^1^Memorial Regional Hospital, Joe DiMaggio Children's Hospital, Hollywood, FL, United States; ^2^Department of Biomedical Engineering, Florida International University, Miami, FL, United States

**Keywords:** ECM, repair, regeneration, cardiovascular, scaffolds

The native extracellular matrix (ECM) is an active material which engages in multi-faceted and complex interactions with cells. Directed cell to ECM communications are known to be essential for functional tissue regeneration such that stem cells, whether native or therapeutically introduced, require an ECM for sensical structural development. Current technologies available for therapeutic interventions in cardiovascular disease have relied largely on mechanical products, xenografts, or some form of composite structure (Bioprosthetics). These products unlike native, biologically living tissues such as heart valves and blood vessels have a shortened life span because of a lack of self-repair and immune mediated destruction. These materials also do not grow with younger patients. Extracellular matrix as a medical therapeutic product involves decellularization of a tissue such that all that remains is the ECM framework, devoid of cellular structures or biological markers. Such scaffolds have enormous potential as a therapeutic option. A breakthrough in regenerative medicine would revolutionize the field of cardiovascular medicine and surgery, where a large sub-set of patients currently face limited treatment options. The advent of regenerative therapies could yield needed, life-saving treatment of congenital cardiovascular defects (e.g., valvular malformations) by supporting somatic growth in the young patient, aid in induction of neovascularization at sites of cardiac damage, and provide hemodynamically resilient, living vascular grafts to replace large arteries that have limited patency due to disease. This Research Topic, aptly named, “Extracellular Matrix for Cardiovascular Reconstruction,” is being presented at this specific time because of the recent explosion of exciting *in vitro, in vivo* and *clinical* findings in regenerative medicine. A total of 8 contributions which includes two mini-reviews, two reviews and four original research articles are summarized here:

In a mini-review article, Delfín et al. remind us of the critical roles that selected ECM proteins play in both cardiovascular homeostasis and in pathologies. Specifically, they focus on a recently identified protein, ABI3BP, that is present in the cardiovascular tissues and whose concentrations are decreased in diseased conditions such as dilated cardiomyopathy. The article further emphasizes the multi-faceted and complex role that a single ECM protein, i.e., ABI3BP, has in both augmenting and reducing cardiac repair/regeneration. The other mini-review by Ramaswamy et al. sheds light on several matricellular proteins such as Decorin, Fibulin-1 and Lumican in the context of vascular tissue engineering. The article describes matricellular protein activity that could positively influence important properties in functional *de novo* blood vessels, such as mechanical integrity, anti-thrombogenicity, endothelialization, and measured recruitment of vascular smooth muscle cells.

Hostile vs. healing macrophage responses in tissue remodeling has long been an aggressively pursued area among cardiovascular researchers. O'Rourke et al. provide a comprehensive review of not only traditional understanding of macrophages in cardiovascular tissues but infuse the article with a report of contemporary findings regarding the restriction of inflammatory responses by macrophages that lead to abnormal ECM remodeling and subsequent heart failure following myocardial infarction. Meanwhile, the other review article by Pattar et al. also targets the critical problem of poor ECM remodeling following myocardial infarction, and discusses the surgical repair of the disease-induced, damaged cardiac muscle, using decellularized ECM bioscaffolds. Of primary interest is the bio-active molecules that are contained within these bioscaffold materials and that can enhance healthy, cardiac regenerative processes while minimizing abnormal tissue remodeling.

Finally, four original research articles by Bax et al.; Gonzalez et al.; Bibevski et al.; Allen et al. round-up the list of contributions in this Research Topic, with all four having a materials and/or mechanobiological framework but in different settings. Following *in vivo* observations, Bax et al. developed an *in vitro* engineered tissue model system to study the individual and combined effects of TGFβ growth factor and cyclic strain on human epicardium-derived cells. Through TGFβ/ALK5-dependent signaling, these cells promoted remodeling of the cardiac ECM via the production of type I collagen. Gonzalez et al. utilized a bioreactor platform to identify how physiologically-relevant, fluid-induced oscillatory shear stresses can differentiate human bone-marrow derived mesenchymal stem cells seeded on a 3-dimensional substrate. The experiments led to ECM formation that exhibited a healthy valve phenotype, which could potentially accelerate heart valve regeneration *in vivo*. Such an approach may serve useful for a heart valve replacement strategy that supports somatic tissue growth in the treatment of critical congenital valve diseases in the young. In the case report with histologic study, Bibevski et al. showed that ECM implanted in the pulmonary artery to fill a defect remodels to mimic the surrounding tissue and the intended biologic function. Clinical reports such as this give us guidance as to what works with bare ECM and what doesn't. Equally relevant is the insightful article by Allen et al. that follows the clinical remodeling of small intestine submucosa (SIS)-ECM after endarterectomy, thereby demonstrating the utility of biological materials in cardiovascular repair procedures.

In summary, understanding the cellular mechanisms of pathological and healthy cardiovascular ECM remodeling under biochemical and/or biomechanical environments of pertinence, as well as the advancement of ECM-mimicking biomaterials for use in cardiovascular surgical repair hold promise for a breakthrough in overcoming current treatment limitations. The 8 articles presented herein have addressed this subject matter, with an appreciation of the state-of-the-field and the current challenges that await us. The topic editors are sincerely appreciative to the 47 authors who contributed to these 8 articles, which will help orient the research community on the ECM, to its properties/responses, and with the overall goal of improving existing treatments for cardiovascular disease. For now, the holy grail of replacing cardiovascular structures with ECM scaffold guided, biologically remodeling tissues that can grow with the patient although within sight, remains elusive.

## Author Contributions

SB, SR, and JH topic editors for this Research Topic, entitled “Extracellular Matrix for Cardiovascular Reconstruction” were responsible for the writing of this editorial. All authors contributed to the article and approved the submitted version.

## Conflict of Interest

The authors declare that the research was conducted in the absence of any commercial or financial relationships that could be construed as a potential conflict of interest.

